# Expression screening using a Medaka cDNA library identifies evolutionarily conserved regulators of the p53/Mdm2 pathway

**DOI:** 10.1186/s12896-015-0208-y

**Published:** 2015-10-08

**Authors:** Ping Zhang, Anne Sophie Kratz, Mohammed Salama, Seham Elabd, Thorsten Heinrich, Joachim Wittbrodt, Christine Blattner, Gary Davidson

**Affiliations:** Institute of Toxicology and Genetics, Karlsruhe Institute of Technology, 76021 Karlsruhe, Germany; Faculty of Biosciences, University of Heidelberg, 69120 Heidelberg, Germany; Department of Anti-Aging Medicine, University of Tokyo, Tokyo, 113-8865 Japan; Department of Developmental Biology and Physiology, University of Heidelberg, 69120 Heidelberg, Germany; Present address: Ludwig Institute for Cancer Research, University of Oxford, Old Road Campus Research Building, Oxford, OX3 7DQ UK; Present address: Cell Cycle Control and Carcinogenesis, German Cancer Research Center (DKFZ), Im Neuenheimer Feld 242, 69120 Heidelberg, Germany

**Keywords:** p53, Mdm2, Gene regulation, Overexpression-screen, Medaka cDNA library

## Abstract

**Background:**

The p53 tumor suppressor protein is mainly regulated by alterations in the half-life of the protein, resulting in significant differences in p53 protein levels in cells. The major regulator of this process is Mdm2, which ubiquitinates p53 and targets it for proteasomal degradation. This process can be enhanced or reduced by proteins that associate with p53 or Mdm2 and several proteins have been identified with such an activity. Furthermore, additional ubiquitin ligases for p53 have been identified in recent years. Nevertheless, our understanding of how p53 abundance and Mdm2 activity are regulated remains incomplete. Here we describe a cell culture based overexpression screen to identify evolutionarily conserved regulators of the p53/Mdm2 circuit. The results from this large-scale screening method will contribute to a better understanding of the regulation of these important proteins.

**Methods:**

Expression screening was based on co-transfection of H1299 cells with pools of cDNA’s from a Medaka library together with p53, Mdm2 and, as internal control, Ror2. After cell lysis, SDS-PAGE/WB analysis was used to detect alterations in these proteins.

**Results:**

More than one hundred hits that altered the abundance of either p53, Mdm2, or both were identified in the primary screen. Subscreening of the library pools that were identified in the primary screen identified several potential novel regulators of p53 and/or Mdm2. We also tested whether the human orthologues of the Medaka genes regulate p53 and/or Mdm2 abundance. All human orthologues regulated p53 and/or Mdm2 abundance in the same manner as the proteins from Medaka, which underscores the suitability of this screening methodology for the identification of new modifiers of p53 and Mdm2.

**Conclusions:**

Despite enormous efforts in the last two decades, many unknown regulators for p53 and Mdm2 abundance are predicted to exist. This cross-species approach to identify evolutionarily conserved regulators demonstrates that our Medaka unigene cDNA library represents a powerful tool to screen for these novel regulators of the p53/Mdm2 pathway.

**Electronic supplementary material:**

The online version of this article (doi:10.1186/s12896-015-0208-y) contains supplementary material, which is available to authorized users.

## Background

The p53 protein is an important tumor suppressor protein and is mutated in about 50 % of human cancers [1]. Mice with a genetic disruption of the p53 gene develop tumors at a particularly young age [2]. One of the major functions of p53 is to control cell proliferation through cell cycle arrest and apoptosis under conditions where there is an increased risk of mutagenesis, for example after DNA damage or nucleotide depletion [3]. For its anti-proliferative activity, p53 uses two distinct cellular pathways. As a transcription factor, p53 enhances the expression of pro-apoptotic target genes and represses transcription of anti-apoptotic target genes [4]. In addition, it associates with pro- and anti-apoptotic proteins in the cytoplasm and controls their function [5].

Under normal growth conditions, p53 is rapidly degraded by the 26S proteasome machinery [6]. This process is mainly controlled by the E3 ubiquitin ligase Mdm2 which mediates polyubiquitination of p53 and promotes the association of p53 with the proteasome [6, 7]. Other E3 ubiquitin ligases have been described to substitute for Mdm2 under particular conditions (reviewed in: [6]). The *mdm2* gene is itself a transcriptional target of p53, thus forming a regulatory feed-back loop with p53. Other proteins like p14^ARF^, the ribosomal protein L11, the FK605-binding protein 25 or KAP1/TRIM28 can impinge on this feedback loop and modulate Mdm2 activity (reviewed in: [6]). Under conditions of an increased risk of carcinogenesis, this feedback loop is interrupted and the normally short half-life of the p53 protein is considerably extended. This results in a strong accumulation of p53, which, together with post-translational modifications that activate p53, leads to cell cycle arrest or apoptosis ([reviewed in: [6]).

Since p53 induces cell death after its activation, significant research efforts are focused on harnessing this activity for tumor therapy, at least for those tumors with wild type p53. This approach, however, requires a more detailed understanding of the regulation of p53.

In order to increase our understanding of the regulation of p53 we searched for novel, evolutionary conserved regulators of p53 and Mdm2 abundance by performing a mammalian cell-based overexpression screen using a cDNA library obtained from the teleost Medaka. In order to facilitate the initial screening process, pools of twenty-four cDNA clones from the master library were prepared as transfection-ready plasmid DNA mixtures. More than one hundred hits were identified in the first round of screening (primary screen) using these cDNA library pools. We sub-screened several of these hits and were able to successfully identify a number of proteins that were not previously known to regulate p53 and/or Mdm2 abundance. The human homologues of several of these genes were shown to regulate p53 and Mdm2 abundance in the same way as the Medaka genes, demonstrating that the identified genes are indeed regulators of p53 and/or Mdm2 abundance.

## Results

In order to find novel regulators of p53 and Mdm2, we screened a cDNA library from the teleost Medaka. The library was prepared from stage eighteen (neurula), stage twenty-four (beginning of neurogenesis) and stage thirty-two (completion of organogenesis) Medaka embryos [8]. It contains almost eighteen thousand full lengths genes of which almost fourteen thousand are annotated. In addition, about three thousand five hundred full length cDNA clones with only partial sequence information are contained in the library. During library preparation, only the most complete representative of each gene was selected, resulting in a unigene cDNA library. In order to simplify the screening procedure, which involved labor-intensive SDS-PAGE and Western Blot analysis of cellular lysates, pools of twenty-four clones were prepared as transfection ready plasmid DNA samples. Over seven hundred such cDNA pools were arrayed in 96-well plates for the primary transfection-based screening experiments.

**Fig. 1:**
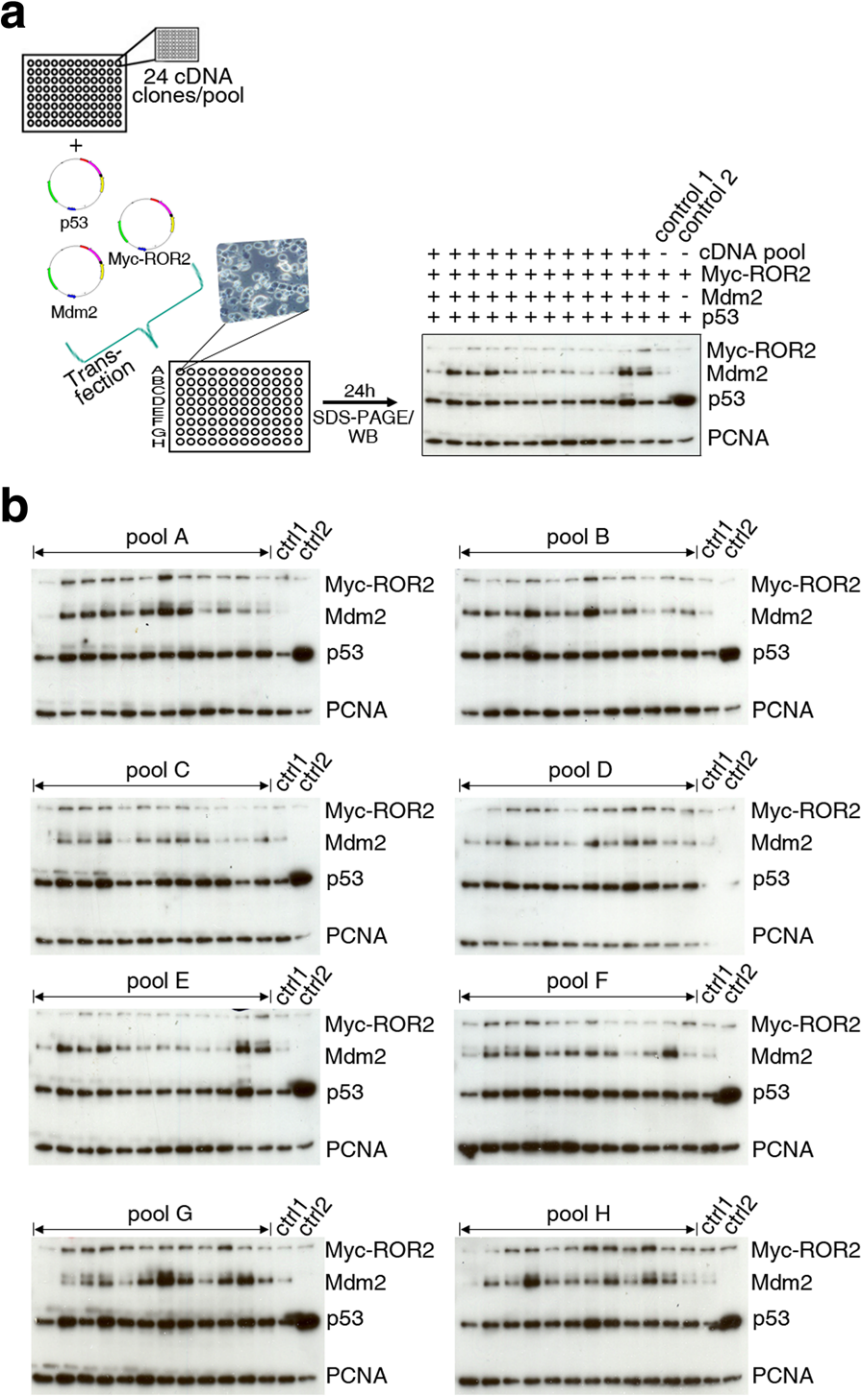
Screening of a Medaka cDNA library. **a** Schematic drawing of the screening process. 24 clones of the cDNA library were pooled and all pools were arrayed in 96-well plates. P53-negative H1299 cells were transfected in 96 well-plates with *p53* (5 ng), *mdm2* (45 ng), one of the cDNA library pools (150 ng) and *myc*-*ror2* (5 ng) as control. To monitor Mdm2 activity, and Mdm2 and p53 abundance in the absence of co-transfected pools of the cDNA library, 2 out of 14 wells were transfected with *p53* and *myc-ror2* (control 2) or with *p53* and *mdm2* and *myc-ror*2 (control 1) without library pools. For transfection, total amounts of plasmid DNA in all samples were adjusted to 205 ng using vector DNA. 24 h after transfection, cells were lysed and abundance of p53, Mdm2 and Myc-ROR2 were determined by Western Blotting. Abundance of PCNA was monitored for loading control. **b** H1299 cells were transfected with plasmids encoding p53, Mdm2 and Myc-ROR2 together with the indicated pools of the cDNA library, without the library (ctrl 1) or without the library and without the plasmid encoding Mdm2 (ctrl 2), for control. 24 h after transfection, cells were lysed and abundance of p53, Mdm2, Myc-ROR2 and PCNA were determined by Western Blotting

Each pool of cDNAs from the library was co-transfected into p53-negative H1299 cells together with *p53* and *mdm2*. The absolute levels and ratios of overexpressed p53 and Mdm2 were optimized to allow both increases and decreases in their abundance to be detected. To monitor Mdm2 activity as well as Mdm2 and p53 abundance in the absence of co-transfected cDNAs from the library, control transfections using only *p53* or *p53* and *mdm2* were performed. As an additional control to determine whether cDNA library pools have a general impact on protein turnover we also transfected the Wnt-receptor protein *ror2* (Fig. 1). Twenty-four hours post-transfection total cell lysates were prepared and the amount of p53, Mdm2 and ROR2 proteins was determined by SDS-PAGE and Western Blotting analysis (Fig. 1). Each library pool was analyzed at least twice and only pools that specifically altered the abundance of co-expressed p53 and/or Mdm2 in at least two experiments were considered as candidates. Using this primary screening approach, we identified one hundred and six pools that either increased or decreased abundance of p53 or Mdm2 or both (Table 1). The majority of the hits increased the abundance of either p53 alone or of p53 and Mdm2, while a lower number of library pools resulted in decreased p53 and/or Mdm2 abundance.

**Table 1:** Summary of the screening of a Medaka cDNA library for evolutionarily conserved regulators of p53 and Mdm2. The table shows the number of hits and their activity on the abundance of p53and/or Mdm2

Property	Number of hits
p53 abundance is decreased	6
p53 abundance is increased	38
Mdm2 abundance is decreased	3
Mdm2 abundance is increased	11
p53 and Mdm2 abundance is decreased	1
p53 and Mdm2abundance is increased	43
p53 abundance is increased, Mdm2 abundance is decreased	1
p53 abundance is decreased, Mdm2 abundance is increased	3
Total	106

In order to confirm that the complexity of the primary screening conditions would not prevent identification of p53 modulators, we spiked a non-modifying (control) pool from the library with plasmid DNA encoding the known p53 modifiers p14^ARF^ and USP7 (see Additional file 1). P14^ARF^ stabilizes p53 by preventing the Ubiquitin-mediated degradation of p53 by Mdm2 [9] and the ubiquitin-specific protease USP7 deubiquitinates both Mdm2 and p53, resulting in their stabilization [10]. Co-expression of *p14*^*arf*^ and *usp7* in amounts similar to those of the individual clones present in the screened pools (about 6 ng) resulted in the expected stabilization of p53 and Mdm2, confirming the suitability of the primary screening conditions (see Additional file 1). Indeed, one of the candidate pools identified in the primary screening contains the Medaka *usp7* gene homologue. The Medaka *p14*^*arf*^ homologue was however not present in the library.

**Fig. 2:**
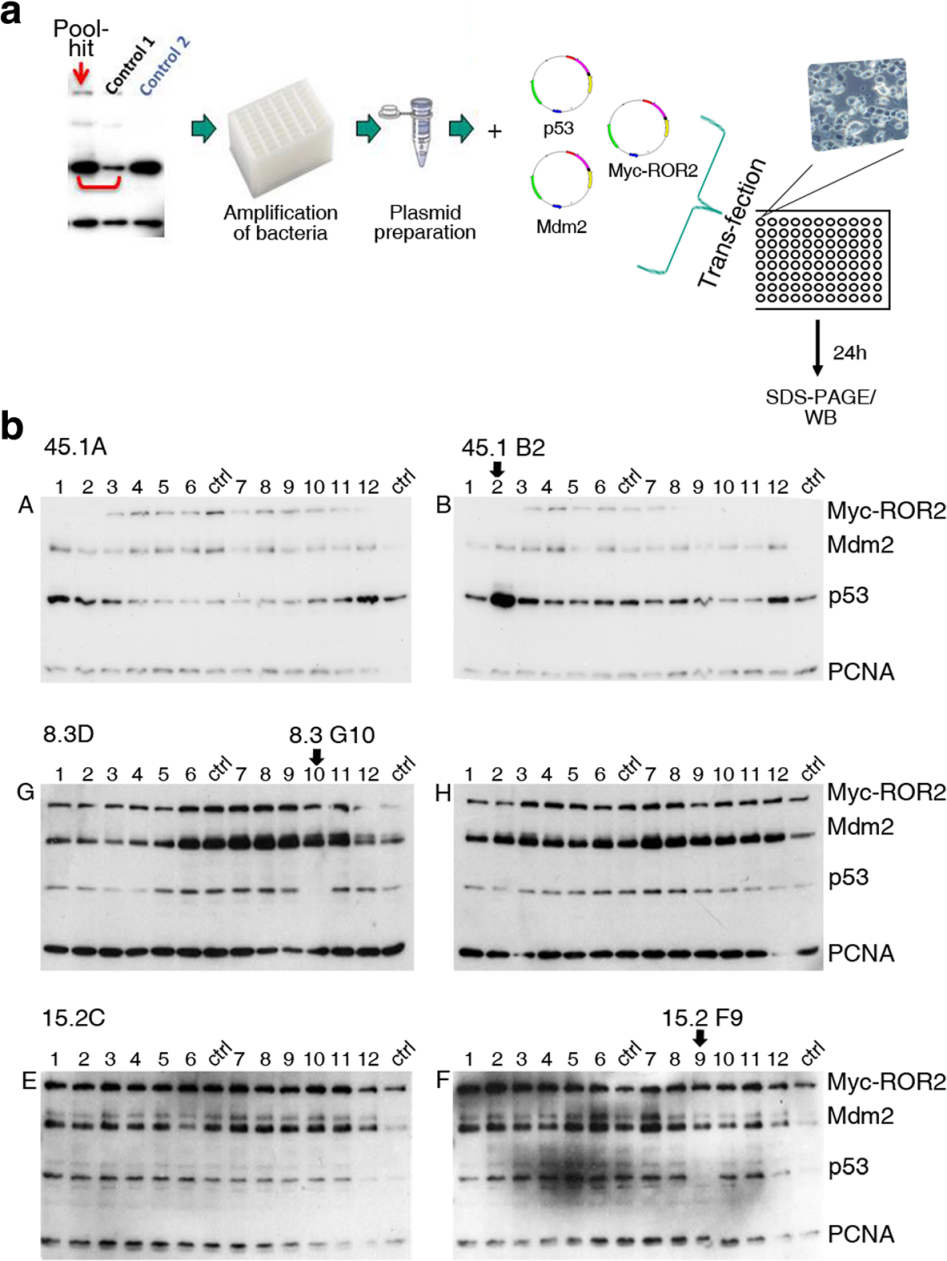
Subscreening of the Medaka cDNA library. **a** From the cDNA pools that were considered to contain regulators of p53 and/or Mdm2, the individual bacteria were amplified from the master library. The plasmids were purified and 12.5 ng of the individual cDNAs were transfected together with *p53* (5 ng), *mdm2* (45 ng) and *myc-ror2* (5 ng) into H1299 cells in a 96-well format. 24 h after transfection, cells were analyzed by Western Blotting. **b** H1299 cells were transfected with plasmids encoding p53, Mdm2 and Myc-ROR2 together with the individual clones of the indicated pool hit or with plasmids encoding p53, Mdm2 and Myc-ROR2 without cDNA clones, for control (ctrl). 24 h after transfection, cells were harvested and analyzed as described in the legend to Fig. 1

In order to identify which genes in the candidate pools were responsible for regulating p53 and/or Mdm2 abundance, we performed a secondary screen using the twenty-four single cDNA clones contained within each candidate pool. The individual cDNA clones were amplified from the arrayed master library and, like the primary screen, were transfected together with *p53*, *mdm2* and *ror2* (Fig. 2a). Each cDNA was analyzed at least twice and only those clones that altered p53 and/or Mdm2 abundance in at least two experiments were considered as candidates. From the one hundred and six pool hits that were identified in the first round of screening, the secondary screening procedure was performed on twenty. The majority (eleven) of these twenty pools harbored a single regulator of p53 or p53 and Mdm2, two pools harbored two regulators and, strikingly, one pool harbored three apparent regulators of p53 and Mdm2 (Fig. 2, Table 2). Thus, overall, 70 % of candidate pools identified and selected for secondary screening were found to contain regulators of p53 and/or Mdm2. Six of the identified regulators affected only p53 and two of the cDNAs affected only Mdm2 abundance. Four of the identified novel regulators increased Mdm2 abundance and decreased p53 abundance and six increased both p53 and Mdm2 abundance (Table 2). Among the seventeen cDNAs that we found to regulate p53 or p53 and Mdm2, twelve were annotated and five had unknown sequences. The molecular function of the genes that we found in the sub-screen (Table 2) ranged from a putative MAPK-activating protein (C1ORF144/SZRD11, [11]) to a lysosomal protein that is involved in the catabolism of heparin and keratan sulphate (GNS; [12]).

**Table 2:** Regulators of p53 and Mdm2 identified by subscreening of the Medaka cDNA library. The table shows the number of the clone in the library, the identification number of the Ensembl database (Ensmble ID), the name of the gene, further details about the protein and its activity within the p53/Mdm2 circuit

Clone number	Ensembl ID	Gene name		Activity towards p53 and Mdm2
2.2 B6	not annotated	unknown		p53 abundance was increased
5.3D G3	ENSORLG00000007935	*q3v605_oryla*	Hoxc8a protein	p53 abundance was increased
5.3D G10	ENSORLG00000008784	*galntl*1	UDP-N-acetyl-alpha-D-galactosamine:polypeptide N-acetylgalactosa-minyltransferase-like 1	p53 and Mdm2 abundance were increased,
6.3 H5	ENSORLG00000003540	*fubp1*	far upstream element-binding protein 1	p53 abundance was decreased, Mdm2 abundance was increased
8.3D G10	ENSORLG00000006197	*rbm15*	RNA binding motif protein 15	p53 abundance was decreased, Mdm2 abundance was increased
9.1 H11	ENSORLG00000001826	*trim25*	Tripartite motif-containing protein 25	p53 and Mdm2 abundance were increased
10.4 B10	ENSORLG00000009742	*bhlhe23*	Basic helix-loop-helix family member e23	p53 abundance was increased
10.4 B12	ENSORLG00000009430	*srsf4*	Serine/arginine-rich splicing factor 4	p53 abundance was increased
15.1 F10	FOE002-P00040-DPE-F_A21	unknown		p53 abundance was decreased, Mdm2 abundance was increased
15.2C F9	not annotated	unknown		p53 abundance was decreased, Mdm2 abundance was increased
15.3 G5	ENSORLG00000016835	*gns*	Glucosamine (N-acetyl)-6-sulfatase	p53 and Mdm2 abundance were increased
15.4 C4	ENSORLG00000004985	*mex3c*	Mex-3 homolog C	p53 abundance was increased
18.2 F5	ENSORLG00000000208	*c1orf144*	C1-open reading frame 144 (PM20, PM21)	p53 abundance was increased
29.3 F1	ENSORLG00000004520	*hsd1 1b 1 l*	Hydroxysteroid (1 l-beta) dehydrogenase 1-like	p53 and Mdm2 abundance were increased
36.4 B D5	not annotated	unknown		p53 and Mdm2 abundance were increased
36.4 B D6	not annotated	unknown		Mdm2 abundance was increased
45.1 B2	ENSORLG00000001401	*fam83f*	Family with sequence similarity 83, member F	p53 abundance was increased

**Fig. 3:**
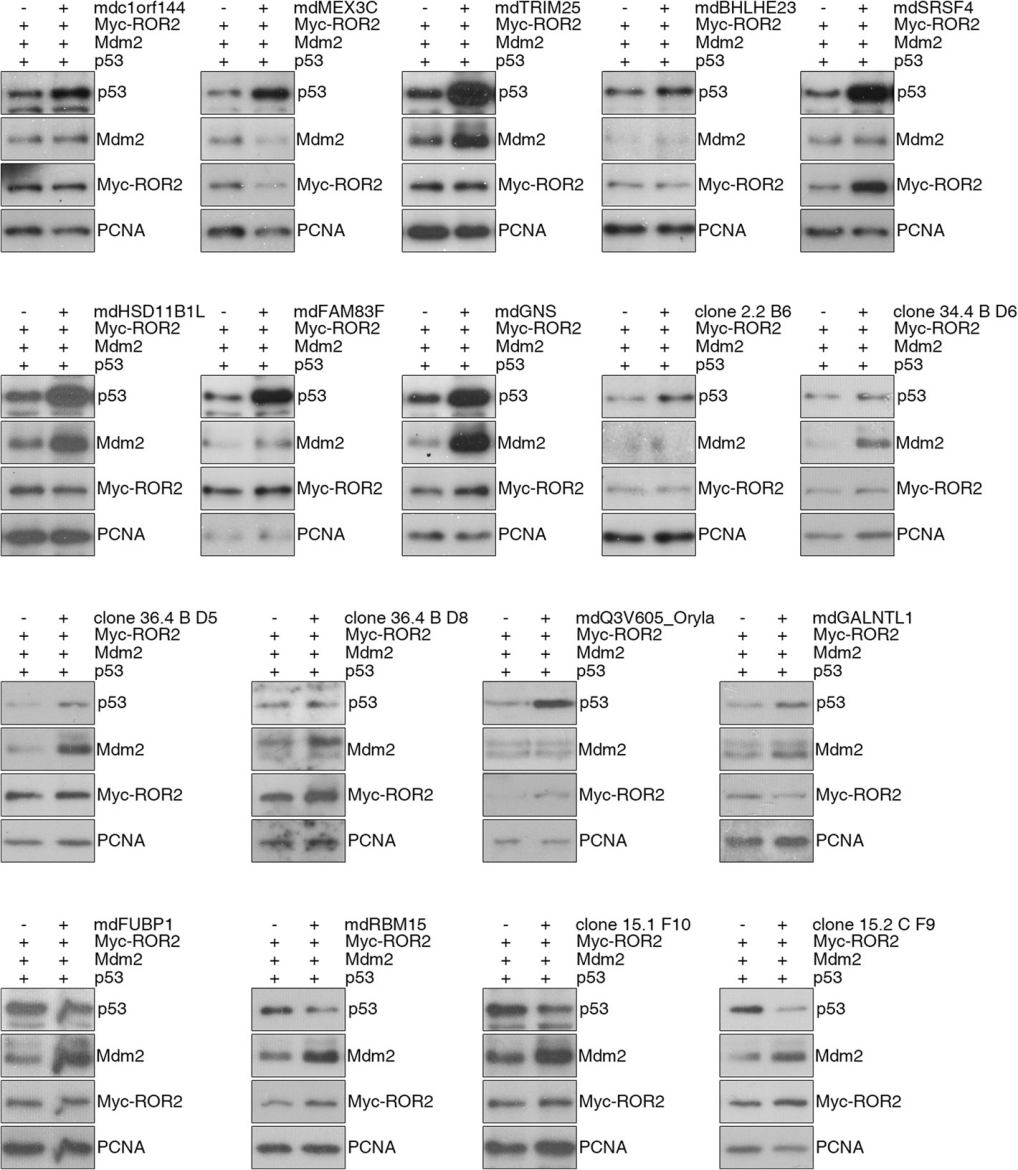
cDNAs identified in a screen using a cDNA library from Medaka induced mammalian p53 and Mdm2. H1299 cells were transfected with the indicated cDNAs together with plasmids encoding p53, Mdm2 and Myc-ROR2. For control, cells were transfected only with plasmids encoding p53, Mdm2 and Myc-ROR2. 24 h after transfection, cells were harvested and analyzed as described in the legend to Fig. 1

Several of the cDNAs identified in the secondary screen were again transfected into H1299 cells to confirm that they indeed regulate the levels of p53 and/or Mdm2. All of the identified Medaka clones regulated p53 and/or Mdm2 (Fig. 3), confirming the result from the secondary screen.

**Fig. 4:**
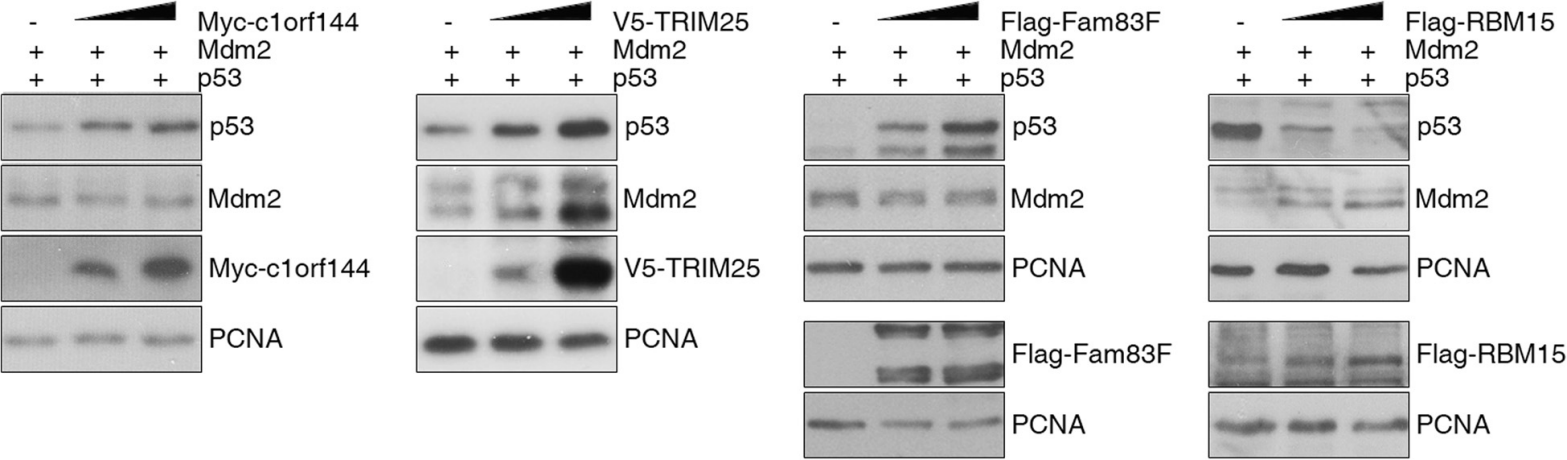
Human homologs of the cDNAs that were identified by screening a Medaka cDNA library control p53 and Mdm2 abundance. H1299 cells were transfected with increasing amounts of plasmids encoding human Myc-tagged C1ORF144, human V5-tagged TRIM25, human Flag-tagged FAM83F and human Flag-tagged RBM15 together with plasmids encoding p53 and Mdm2. For control, cells were transfected only with plasmids encoding p53 and Mdm2. 24 h after transfection, cells were harvested and analyzed as described in the legend to Fig. 1

We have performed a cell culture-based cross-species expression screen where a cDNA library from Medaka was used to screen for regulators of human p53 and mouse Mdm2 in human cells. One aim of this screen was to identify evolutionarily conserved modifiers of the p53 signaling pathway. Considering the evolutionary distance of teleosts and mammals we tested whether the human orthologues of the identified Medaka genes would also be able to regulate p53 and Mdm2. As shown in Fig. 4, the human orthologues of some of the genes identified (e.g. *c1orf144*, *trim25*, *fam83f* and *rbm15*) also regulated p53 and/or Mdm2 abundance (Fig. 4). C1ORF144/SZRD1, increased p53 abundance, TRIM25 increased p53 and Mdm2 abundance, FAM83F increased p53 abundance and decreased Mdm2 abundance and RBM15 increased Mdm2 abundance and decreased p53 abundance. We also tested the ability of C1ORF144/SZRD1 and FAM83F to regulate endogenous p53 levels in p53-positive cells and in each case we could see the expected effect (see Additional file 2). Furthermore, we recently demonstrated that TRIM25 also regulates endogenous p53 [13]. These results demonstrate that cell culture-based expression screening using a cDNA library from Medaka can identify evolutionarily conserved regulators of p53 and Mdm2.

## Discussion

We have performed a cross-species cell-based overexpression screen using a Medaka cDNA library to screen for novel regulators of p53 and Mdm2 in human cells. SDS-PAGE and Western blotting was used as a read–out to specifically detect the abundance of these proteins. Since the cDNA library contained more than seventeen-thousand genes, we used pools of twenty-four individual cDNAs for transfection. Strikingly, we found more than one hundred cDNA pools that altered the abundance of co-transfected p53 and Mdm2 in p53-negative H1299 cells. The strategy of using pooled cDNAs in the primary screen, where only six nanograms of each individual clone was co-expressed with *p53* and *mdm2*, likely resulted in missed hits (i.e. false negatives), due to limits in sensitivity. About two hundred nanograms is the maximum amount of plasmid DNA that can be transfected in 96-wells without significantly increasing cell toxicity and for this reason it was considered counter productive to further increase the amount of library clones in the primary screen. In the secondary screens, where double the amount of individual cDNA clones were transfected compared to the primary screen, a more robust modification of p53 and/or Mdm2 protein levels were indeed seen. Reducing the pool size to twelve rather than twenty-four would likely result in the identification of additional modifiers, however every screening experiment has its particular limitations and we believe that a good compromise was achieved using our strategy. Another reason for missing potential regulators is that the activity of one regulator can be masked by an opposing activity of additional regulators present in the same pool. Although this is unlikely to be a major factor, the surprising identification of three independent regulators in one of the pools confirms that this is an issue worth considering. In spite of these limitations, more than one hundred hits were identified using the library pools, several of which have indeed been verified.

Twenty of the pools identified in the first round of screening were selected for a second round of screening in order to isolate the corresponding p53/Mdm2 modifier. Fourteen of the twenty pools indeed contained at least one cDNA that regulated p53, which represents a success rate of 70 %. One of the clones that we identified as a regulator of p53 and/or Mdm2, the splicing factor FUBP1 (far upstream binding protein 1) is already known to stabilize p53 in response to oxidative stress [14] yet all other regulators of p53 identified in the sub-screen were not previously known to regulate p53 or Mdm2. Due to the relatively well annotated nature of the cDNA library it is possible to scan the library database for the identity of putative regulators within a candidate pool. One of the pools identified in the primary screen was found to harbor the known p53 and Mdm2 regulatory gene, *usp7* [10]. Other known regulators of p53 or Mdm2 like p14^Arf^ do not appear to be present in the library. Nevertheless, as discussed above, it is not possible to find all regulators of p53 and many of the pools identified in the primary screening have yet to be analyzed by secondary screening. Indeed, we only subscreened 20 % of the library. Thus, it is possible that the remaining 80 % of the library will also contain some known regulators of p53 and/or Mdm2 that were not annotated. Finally, this library was prepared from mRNA extracted from developing embryos and early juvenile fish and it is unlikely that all regulators of p53 will be represented.

Our previous success in searching for evolutionarily conserved interactors using cross-species screening methods [15, 16] prompted us to use a full length unigene library from the teleost Medaka to screen for evolutionarily conserved regulators of mammalian p53 and Mdm2. Several of the hits were validated after subsequent analysis of the corresponding human orthologues. Importantly, all human homologs analyzed regulated p53 and Mdm2 in the same way as the Medaka library genes. Thus, although Medaka and humans are evolutionarily highly divergent, the principle mechanism used to regulate p53 levels appears to be highly conserved. The use of a Medaka cDNA library to screen for regulators of human genes is a promising approach in other areas of research. The main challenge will be to characterize the mode of action of the identified regulators.

## Conclusions

Our results show that it is possible to identify regulators of p53 and Mdm2 levels by performing a cross-species, cell-based cDNA library expression screen. Rapid screening of the entire library of over seventeen thousand genes was made possible by preparing pools of library cDNAs that were used in the primary screening method. It is likely that such a screening method can also identify regulators of the abundance of other cellular proteins.

## Methods

### Cell lines and their treatments

H1299 cells (p53-negative) were cultured in DMEM containing 10 % foetal bovine serum (FBS) and 1 % penicillin/streptomycin according to standard conditions. For transfection of the library, 5x10^4^ cells/well were transferred into 96-well plates and transiently transfected with 150 ng DNA from the cDNA library, 5 ng *p53*, 45 ng *mdm2* and 5.5 ng 6x*myc*-*ror2* using PromoFectin (PromoKine) according to the manufacturer’s recommendation. The total amount of transfected cDNA was always kept constant at 205.5 ng for all samples, including controls, and adjusted with empty vector DNA where necessary.

### Medaka cDNA library

The library has been described in [8]. Small aliquots from the individual bacterial clones of the arrayed Medaka library (in 384 well plates) were first transferred to 184 x 96-well plates containing 180 μl LB broth + 10 % glycerol and amplified for 24 h at 37 °C using a high frequency orbital shaker. A total of 17.525 individual clones were amplified in this way, creating a duplicated bacterial library in 96-well format. Bacterial cultures from two adjacent rows of the 96-well plates (24 individual wells) were then pooled (4 ml volume) and used for mini-prep isolation of plasmid DNA (Qiagen kit). A total of 732 pooled plasmid DNA samples were then arrayed in 96-well PCR plates as transfection ready cDNA library pools. 150 ng of these pools were used for the primary transfection-based screening experiments.

### Plasmids

The plasmids encoding *p53* and *mdm2* have been described earlier [7]. *Myc-ror2* was constructed by PCR amplification. First, 6myc-tags were amplified and inserted into pCS2+ at *EcoR1* and *Sac1* sites, followed by insertion of PCR-amplified *ror2* into *Sac1* and *XbaI* sites. *v5-tagged trim25* was amplified by PCR with inclusion of the sequence for the V5-tag in the forward primer using a plasmid for human gfp-trim25 that has been sent to us by Germana Meroni. *myc-tagged c1orf144* was amplified by PCR with inclusion of the sequence for the Myc-tag in the forward primer and reversely transcribed RNA as a template. *flag-tagged fam83f* was amplified by PCR with inclusion of the sequence for the Flag-tag in the forward primer and reversely transcribed RNA as a template. Sequences of cloning primers are available on request. *flag-tagged rbm15s* was provided by Stephen Morris, *p14*^*ARF*^ by Karen Vousden and *usp7* by Wei Gu. The plasmids encoding *mdc1orf144*, *mdmex3c*, *mdtrim25*, *mdbhlhe23*, *mdsrsf4*, *mdhsd11b1l*, *mdfam83f mdgns*, clone *2.2 B6*, clone *34.4 B D6*, clone *36.4B D5*, clone *36.4 B D8*, *mdQ3V605_Oryla*, *mdgalntl1*, *mdfubp1*, clone *15.1 F10*, clone *15.2 C F9* and *mdrbm15* have been obtained directly from the Medaka library.

### Antibodies

The antibodies DO-1 (p53), PC10 (anti-proliferating nuclear cell antigen; PCNA) and 9E10 (Myc) were purchased from Santa Cruz. The anti-V5 antibody was obtained from Serotec and the anti-Flag antibody was from Sigma. HRP-coupled secondary antibodies were purchased from Dako.

### SDS-PAGE and Western blotting

Cells were washed with PBS and lysed in NP40 lysis buffer (50 mM Tris pH 8.0, 150 mM NaCl, 5 mM EDTA, 1 % NP40, 1 mM PMSF) for 10 min on ice. Lysates were centrifuged at 20.000 g for 2 min to remove insoluble material and clarified supernatants were mixed with an equal volume of 2x sample buffer (4 % sodiumdodecylsulfate (SDS), 160 mM Tris pH6.8, 20 % glycerol, 10 % 2-mercaptoethanol, 0.002 % bromophenol blue). The samples were heated at 95 °C for 4 min, loaded onto an SDS-10 % polyacrylamide gel, separated by electrophoresis and transferred onto a polyvinylidene difluoride (PVDF) membrane (Millipore). Membranes were blocked for 30 min in 5 % dry milk and 0.2 % Tween-20 in PBS and incubated overnight with the primary antibodies. After washing 3 times for 5 min in 0.2 % Tween-20 in PBS, the secondary antibody was added and incubated for 1.5 h. After extensive washing, the blots were developed by adding ECL and exposing the membranes against an X-ray film.

## Availability of supporting data

All supporting data are included as supplementary files.

## Additional files

Additional file 1:
**Effect of known modifiers of p53 using the primary screening conditions with cDNA pools.** Western Blot of lysates from H1299 cells transfected as indicated in 96-well format and harvested 30 h post-transfection. In order to create the complexity of the primary screening conditions, 100 ng of a control (non-modifying) cDNA library pool was spiked with 5 ng and 10 ng of genes encoding the known p53 modifiers hP14ARF and hUSP7, and co-transfected with of *p53 (*5 ng) and *mdm2 (*50 ng) (lanes 3–7). As controls a non-functional *USP7* mutant was used, as well as a candidate cDNA library pool containing the *C1orf144* gene encoding szrd1, which wasidentified as a positive hit for p53 stabilisation, (lane 2). (PDF 868 kb) Additional file 2:
**Regulation of endogenous p53 and its target gene Bax after overexpression of c1orf144 or Fam83F.** A. U2OS cells were transfected with a plasmid encoding human C1ORF144 or with vector DNA for control using Promofectin. B. MCF-7 cells were transfected with a plasmid encoding human FAM83F or with vector DNA for control using Promofectin. All transfections were performed with 2 μg of plasmid DNA in 24-well plates. The cells were lysed 24 h after transfection. Abundance of p53 and Bax was monitored by Western blotting. (PDF 325 kb)

## Abbreviations

Mdm2: Mouse double minute 2
cDNA: copy DNA
DNA: Desoxyribonucleic acid
ARF: Alternative reading frame
KAP1: KRAB-associated protein 1
SDS-PAGE: Sodium dodecylsulphate-polyacrylamide gel electrophoresis
USP7: Ubiquitin-specific peptidase 7
MAPK: Mitogen-activated protein kinase
C1ORF144: Chromosome 1-open reading frame 144
SZRD11: SUZ RNA binding domain containing protein 11
GNS: Glucosamine (N-acetyl)-6-sulfatase
TRIM25: Tripartite motif-containing protein 25
FAM83F: Family with sequence similarity member F
rbm15: RNA binding motif protein 15
FUBP1: Far upstream binding protein 1
ng: Nanogram
LB: Luria-Bertoni
C: Celsius
PCR: Polymerase chain reaction
RNA: Ribonucleic acid
MEX3C: Mex-3 RNA binding protein C
BHLHE23: Basic helix-loop-helix family member e23
SRSF4: Serine/arginine-rich splicing factor 4
hsd11b1l: Hydroxysteroid (11-beta) dehydrogenase 1-like
PBS: Phosphate-buffered saline
NaCl: Sodium chloride
NP40: Nonidet P40
PMSF: Phenylmethylsulfonylfluorid
min: Minute(s)
h: Hour(s)
ECL: Enhanced chemiluminescence
PCNA: Proliferating cell nuclear antigen
