# Supplementary material for: Expression screening using a Medaka cDNA library identifies evolutionarily conserved regulators of the p53/Mdm2 pathway
Source: BMC Biotechnol. 2015 Oct 8;15:92. doi: 10.1186/s12896-015-0208-y (PMC4599741; doi:10.1186/s12896-015-0208-y)
Supplement: Additional file 1: — Effect of known modifiers of p53 using the primary screening conditions with cDNA pools. Western Blot of lysates from H1299 cells transfected as indicated in 96-well format and harvested 30 h post-transfection. In order to create the complexity of the primary screening conditions, 100 ng of a control (non-modifying) cDNA library pool was spiked with 5 ng and 10 ng of genes encoding the known p53 modifiers hP14ARF and hUSP7, and co-transfected with of p53 (5 ng) and mdm2 (50 ng) (lanes 3–7). As controls a non-functional USP7 mutant was used, as well as a candidate cDNA library pool containing the C1orf144 gene encoding szrd1, which wasidentified as a positive hit for p53 stabilisation, (lane 2). (PDF 868 kb) [file 12896_2015_208_MOESM1_ESM.pdf]

p53 (5 ng)	+	+	+	+	+	+	+	+	+
mdm2 (50 ng)	+	+	+	+	+	+	+	+	+
candidate cDNA pool (100 ng)		+							
control cDNA pool (100 ng)			+	+	+	+	+	+	+
arf				5	10				
usp7						5	10		
mutant *usp7*								5	10

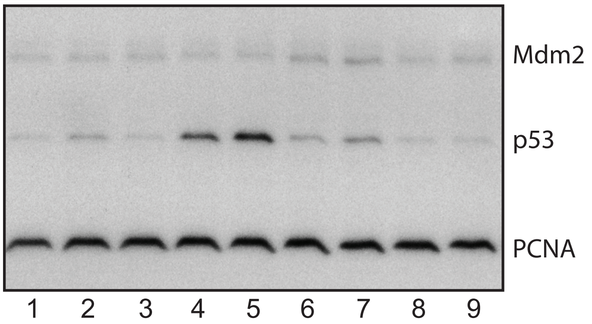

Supplementary Figure S1. Effect of known modifiers of p53 using the primary screening conditions with cDNA pools. Western Blot of lysates from H1299 cells transfected as indicated in 96-well format and harvested 30 hours post-transfection. In order to create the complexity of the primary screening conditions, 100 ng of a control (non-modifying) cDNA library pool was spiked with 5 ng and 10 ng of genes encoding the known p53 modifiers hP14ARF and hUSP7, and co-transfected with of *p53 (*5 ng) and *mdm2 (*50 ng) (lanes 3-7). As controls a non-functional *USP7* mutant was used, as well as a candidate cDNA library pool containing the *C1orf144* gene encoding szrd1, which was identified as a positive hit for p53 stabilisation, (lane 2).
