# Supplementary material for: Expression screening using a Medaka cDNA library identifies evolutionarily conserved regulators of the p53/Mdm2 pathway
Source: BMC Biotechnol. 2015 Oct 8;15:92. doi: 10.1186/s12896-015-0208-y (PMC4599741; doi:10.1186/s12896-015-0208-y)
Supplement: Additional file 2: — Regulation of endogenous p53 and its target gene Bax after overexpression of c1orf144 or Fam83F. A. U2OS cells were transfected with a plasmid encoding human C1ORF144 or with vector DNA for control using Promofectin. B. MCF-7 cells were transfected with a plasmid encoding human FAM83F or with vector DNA for control using Promofectin. All transfections were performed with 2 μg of plasmid DNA in 24-well plates. The cells were lysed 24 h after transfection. Abundance of p53 and Bax was monitored by Western blotting. (PDF 325 kb) [file 12896_2015_208_MOESM2_ESM.pdf]

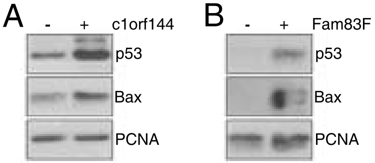

Regulation of endogenous p53 and its target gene Bax after overexpression of c1orf144 or Fam83F. **A.** U2OS cells were transfected with a plasmid encoding human C1ORF144 or with vector DNA for control using Promofectin. **B.** MCF-7 cells were transfected with a plasmid encoding human FAM83F or with vector DNA for control using Promofectin. All transfections were performed with 2 µg of plasmid DNA in 24-well plates. The cells were lysed 24h after transfection. Abundance of p53 and Bax was monitored by Western blotting.
